# The mean cell volume difference (dMCV) reflects serum hypertonicity in diabetic dogs

**DOI:** 10.1371/journal.pone.0219864

**Published:** 2019-07-23

**Authors:** Olga C. Norris, Thomas Schermerhorn

**Affiliations:** Department of Clinical Sciences, College of Veterinary Medicine, Kansas State University, Manhattan, Kansas, United States of America; Medical University of Vienna, AUSTRIA

## Abstract

Serum hypertonicity may develop during diabetes mellitus due to hyperglycemia and other biochemical changes. Hypertonicity may produce detrimental cellular and systemic effects and has been identified as a serum marker for some clinical disorders. In non-diabetic dogs, the mean cell volume difference, a novel erythrocyte measure, is increased by serum hypertonicity. However, it is not known whether hyperglycemic hypertonicity produces a similar change. The hypothesis that the mean cell volume difference could detect serum hypertonicity in diabetes was investigated in a group of thirty-two dogs with naturally-occurring diabetes mellitus that were prospectively recruited over a 1-year period from the outpatient population of a veterinary teaching hospital. The effect of hyperglycemia on the mean cell volume difference and the ability of the mean cell volume difference to predict serum hypertonicity were examined. Serum hyperosmolality and hypertonicity due to hyperglycemia was present in 91% and 94% of dogs, respectively. Hyperglycemia was the principal cause identified for serum hypertonicity and hyperosmolality. Using a cut-off value of ≥ 3 μm^3^ for the mean cell volume difference, serum hypertonicity ≥ 320 mmol/kg was identified with 79% sensitivity and 61% specificity. The dMCV correlated with changes in serum glucose, tonicity, and measured osmolality. Dogs with a mean cell volume difference ≥ 3 μm^3^ were at risk for serum tonicity ≥ 320 mmol/kg (risk ratio = 2.2) and serum glucose ≥ 13.9 mmol/L (risk ratio = 2.3). In conclusion, the mean cell volume difference is a useful surrogate marker for detecting serum hypertonicity in diabetic dogs and elevated mean cell volume difference is associated with increased risks for clinically relevant serum hypertonicity and hyperglycemia.

## Introduction

Hypertonicity is defined by an elevated serum concentration of effective osmoles and is a well described clinical feature of certain disorders, such as ethylene glycol toxicity or conditions that cause hypernatremia. Tonicity, a physiochemical property of serum, is determined by the concentration of effective osmoles that influence water movement across semi-permeable cell membranes [1]. Electrolytes and glucose are the principal effective osmoles present in serum of healthy dogs. The ability of glucose to act as an effective osmole in serum is enhanced in the absence of insulin, which impairs cellular glucose uptake and ‘traps’ glucose in the extracellular compartment. Tonicity cannot be directly measured by any convenient method but is approximated in healthy dogs by the serum total osmolality, which can be measured using laboratory methods. Tonicity is maintained within strict physiologic limits with little day-to-day variation within individuals or within members of the same species [2, 3]. Under most circumstances, serum tonicity, also known as effective osmolality, is a major component of the serum total osmolality. In normal dogs, tonicity is maintained around 295 mOsm/L and accounts for approximately 98% of the serum total osmolality, which is maintained around 300 mOsm/L. Permeable serum osmoles, such as urea, that freely move across cell membranes contribute to the calculated or measured total osmolality but do not contribute to serum tonicity [2].

A variety of human and animal disorders are associated with changes in serum tonicity [4, 5, 6, 7, 8]. Serum hypertonicity may serve as an early marker for several human disease states, including diabetes [9, 10, 11]. Major clinical manifestations of serum hypertonicity are exerted via osmotic effects on cellular function but other mechanisms that may produce cellular dysfunction have been described, including altered protein synthesis and function, altered cell membrane function, and DNA damage [12, 13, 14].

Serum hypertonicity *per se* is an infrequently discussed aspect of diabetes mellitus but there is evidence that it influences multiple aspects of diabetes pathology. The relationship between hypertonicity and diabetes is likely to be complex. An epidemiologic study showed that pre-diabetic human patients with plasma hypertonicity progress more rapidly to overt diabetes than those with normotonicity [11]. Obesity, a condition that conveys diabetes risk in humans, is also associated with a risk for plasma hypertonicity that is independent of the glucose concentration [10]. While similar roles for hypertonicity in the progression of diabetes in animals have not been demonstrated, several studies have examined tonicity in dogs with clinically overt diabetes. One study reported a high prevalence of serum hypertonicity among dogs with diabetic ketosis before insulin treatment was begun [6] and another found that hyperosmolarity and hypertonicity were common pre-treatment findings in a group of 66 dogs with complicated diabetes [15].

Exploration into possible roles for hypertonicity in canine diabetes is hindered by the lack of a practical clinical parameter to assess tonicity. In healthy dogs, the serum total measured osmolality closely approximates the tonicity. However, the relationship is less predictable in ill dogs with metabolic disturbances. For example, accumulation of ineffective osmoles (e.g. urea) or unmeasured osmoles (e.g. lactate) increases serum total osmolality but may not exert tonic effects [16]. Likewise, in healthy dogs with euglycemia, tonicity is approximated by twice the sodium concentration (doubling the sodium concentration accounts for its accompanying anion) because glucose only contributes about 1.5% of total osmoles in this circumstance. When diabetes is present, hyperglycemia not only contributes to tonicity as an effective osmole but accumulation of large concentrations of glucose in serum may also influence the sodium concentration [6]. Thus, the predictive relationship between serum sodium and tonicity is disrupted on several levels by the onset of hyperglycemia. Lacking a practical method to determine tonicity directly, a surrogate marker that responds to changes in serum tonicity would be useful in the clinical setting. The mean cell volume difference (dMCV) meets these requirements and has been validated as a clinical marker for serum hypertonicity in humans and dogs [17, 18, 19].

The dMCV is a measure of the osmotic disequilibrium that occurs when erythrocytes from hypertonic plasma are exposed to an isotonic buffer used in automated cell counters in the clinical pathology laboratory [17, 20]. The *in vitro* volume changes represented in the dMCV measurement occur because of cellular adaptations, such the accumulation of intracellular metabolites (‘idiogenic osmoles’) that increase intracellular tonicity when RBCs are exposed to a hypertonic plasma environment *in vivo*. A previous study that examined a population of hospitalized dogs with a variety of illnesses showed that the dMCV was significantly larger in dogs with hypertonicity compared to those with normotonicity [18]. For the majority of those dogs, sodium was the serum osmole responsible for hypertonicity, which is consistent with experimental studies showing that the dMCV is sensitive to relatively mild disturbances in serum sodium and osmolality that occur when humans or dogs undergo a period of water deprivation [17, 19]. However, no study has determined whether glucose induces similar changes in the dMCV when hypertonicity is caused by hyperglycemia or whether dMCV is a clinical indicator of serum hypertonicity in dogs with naturally-occurring diabetes. We hypothesized that the dMCV is related to the magnitude of serum hypertonicity caused by hyperglycemia in insulin-treated, clinically stable dogs with naturally-occurring diabetes. To address the hypothesis, the study evaluated 1) relationships between clinical measures (hyperglycemia, hyperosmolarity and hypertonicity) and dMCV and 2) risk relationships between elevated dMCV and metabolic measures that impact serum tonicity.

## Methods

Diabetic dogs from an outpatient population were consecutively enrolled through the 1-year period from 2016–2017. Diabetic dogs presented by the owner for evaluation for any reason to the Veterinary Medical Center of the College of Veterinary Medicine, Kansas State University were eligible for inclusion. Signed owner consent was obtained before dogs could be enrolled into the study. Enrollment was restricted to clinically stable diabetic dogs to avoid the potential confounding influences of untreated hyperglycemia and its accompanying acute metabolic complications on the dMCV measurement. Specific inclusion requirements were: 1) a previous diagnosis of diabetes mellitus; 2) insulin treatment had been given for a least 1 month; 3) the dog was clinically stable and was being maintained as an outpatient. For purposes of study enrollment, diabetic dogs that were maintained at home and received daily insulin injections, displayed normal physical activity, appetite, and water consumption at home, and had no health complaints other than diabetes at the time of enrollment were considered ‘clinically stable’. Newly diagnosed diabetic dogs and diabetic dogs with evidence of complicated diabetes (diabetic ketoacidosis or hyperglycemic hyperosmolar syndrome) or concurrent illness that warranted hospitalization were excluded. Dogs with anemia were excluded to eliminate possible confounding influences of altered RBC cell volume responses or the presence of increased numbers of reticulocytes on dMCV. Each dog had a single blood sample (6 ml) drawn by venipuncture for a complete blood count (CBC), serum biochemistry panel, and serum total osmolality (OsM_T_) measurement. Consistent with our routine recommendations for diabetic dogs, owners had been instructed to adhere to their morning routine schedule for feeding and insulin administration prior to the veterinary visit. The study was performed in accordance with the Kansas State University guidelines for animal research and was reviewed and approved by the Institutional Animal Care and Use Committee (IACUC #3827).

The Clinical Pathology Laboratory at the Kansas State University Veterinary Diagnostic Laboratory performed all laboratory assessments. CBCs and serum biochemistry profiles were completed using an Advia 2120 Hematology System (Siemens Medical Solutions, Inc.; Malvern, PA) and a COBAS C501 Chemistry Analyzer (Roche Diagnostics; Indianapolis, IN). A spun hematocrit, which was used to calculate dMCV, was determined using a card reader and was performed by the clinical pathology laboratory as a routine part of the CBC analysis for each dog. All blood samples for CBC and serum biochemistry profile were analyzed within 24 hours of collection.

Serum osmolality calculations were carried out using measured serum concentrations of Na (mmol/L), K (mmol/L), glucose (mmol/L), and Serum Urea Nitrogen (SUN; mmol/L) concentrations. Calculated total osmolality (OsM_C_; mmol/kg) (Eq 1) and serum tonicity (ST; mmol/kg) (Eq 2) were determined using standard clinical formulas that have been recommended for osmolality determinations in dogs [21].

$$OsM_{C}=2\left(Na+K\right)+SUN+GLUCOSE$$ Eq 1

ST=2(Na+K)+GLUCOSE Eq 2

The dMCV (μm^3^) was determined using measured values for the mean corpuscular volume (MCV_M_; μm^3^), spun hematocrit (hct; %), and red blood cell (RBC) count (x10^12^/L) obtained from the CBC results. The dMCV (Eq 3) was calculated as shown below (Eq 3) using a previously described method [17].

$$dMCV={MCV}_{M}-hct\times 10RBC$$ Eq 3

Serum OsM_T_ was measured using the freezing-point depression method by a benchtop osmometer (Micro OSMETTE, Precision Systems Inc). Serum OsM_T_ determinations were carried out in a single batch using serum samples that had been frozen at –80°C until osmometry was performed. Each sample was measured in triplicate and the separate determinations averaged to provide the final result recorded for analysis. To be accepted for inclusion into the final data set, each of the osmolality determinations had to be within +/- 3 mmol/kg of the mean.

## Data analysis

Summarized clinicopathologic data were expressed as mean +/- standard deviation and median and range. Erythrocyte indices and concentrations of measured serum osmoles were compared to the laboratory reference ranges provided by the Kansas State Diagnostic Laboratory. The individual contribution of each of the measured effective osmoles (Na, K, and glucose) to tonicity was determined by dividing the mean value for each osmole by the mean ST and converting the resulting proportion to a percentage. For simplicity, the percent contributions of “Na” and “K” combine the contribution of the specified cation as well as its non-specified anion. Dogs were classified as having hypertonicity or hyperosmolality, respectively, if ST or OsM_T_ was >320 mOsm/kg. These cut-off values for ST and Osm_T_ represent clinically significant changes in tonicity or osmolarity and have used in an earlier study of dMCV in dogs [18]. Dogs were classified as having clinically relevant hyperglycemia if serum glucose was > 13.9 mmol/L. Dogs with hyperglycemia > 13.9 mmol/L are expected to display clinical signs of diabetes and glucose < 13.9 mmol/L has been recommended as a treatment target for dogs with diabetes mellitus [6]. The cut-off for dMCV was set at > 3 um^3^, a cut-off previously determined to provide optimal sensitivity and specificity for detecting serum hypertonicity of >320 mmol/kg in dogs [18]. Osmolality data was confirmed to be normally distributed using the Kolmogorov-Smirnov test (https://www.socscistatistics.com/tests/kolmogorov/Default.aspx) and the hypothesis that ST was different than OsM_T_ was tested using Student’s T-test for repeated measures (https://www.socscistatistics.com/tests/ttestdependent/Default.aspx). Serum osmole concentrations, ST, OsmT, and dMCV between dogs stratified by level of hyperglycemic were compared using the Mann-Whitney U test (https://www.socscistatistics.com/tests/signedranks/default2.aspx). Correlations between dMCV and serum glucose, ST, and OsM_T_ were tested using Pearson’s Correlation Coefficient and were performed using an online tool (https://www.socscistatistics.com/tests/pearson/default2.aspx) Test characteristics (sensitivity and specificity) were calculated using an online tool (https://www.medcalc.org/calc/diagnostic_test.php). To test the hypothesis that elevated dMCV reflected risk for the presence of glycemic disturbances (serum osmolality and glucose concentrations), risk ratios (RR) were calculated using an online statistical tool (https://www.medcalc.org/calc/relative_risk.php). For RR calculation, a dMCV > 3 μm^3^ was considered the exposure and variables of interest (serum glucose > 13.9 mmol/L, ST > 320 mmol/kg, OsM_T_ > 320 mmol/kg) considered positive outcomes. For all statistical tests, results were considered significant if the p value was < 0.05.

## Results

Summary statistics for serum concentrations of major osmoles (Na, K, glucose, SUN), osmolality assessments (OsM_T_, OsM_C_, and ST) and dMCV from the 32 dogs that met the study inclusion criteria are shown in Table 1. Mean serum Na and K concentrations were within the reference ranges provided by the clinical laboratory, although Na concentrations approximated the lower end of the reference range. Sodium disturbances were present in 37.5% of dogs. Ten dogs had mild hyponatremia (range 139–143 mmol/L) and two had mild hypernatremia (152 and 154 mmol/L). Potassium disturbances were present in 40.6% of dogs; all 13 affected dogs had mild hyperkalemia (range 5.2–5.8 mmol/L). The SUN concentration was within or below the reference range in all dogs except for two dogs with slight increases (10.7 and 15.7 mmol/L).

**Table 1:** Summary statistics for diabetic dogs (n = 32) included in the study group.

	Mean	SD	Median	Range	Reference Range
**Sodium (Na)**	145.7	3.5	145.5	139–154	144–151 mmol/L
**Potassium (K)**	4.8	0.48	4.8	3.9–5.8	3.7–5.0 mmol/L
**Serum Urea Nitrogen (SUN)**	7.0	3.0	6.25	2.5–15.7	2.9–10.4 mmol/L
**Glucose**	15.8	8.24	14.3	3.2–29.1	3.9–6.7 mmol/L
**OsM**_**T**_	323.8* †	14.4	321	302–376	295–305 mmol/kg
**OsM**_**C**_^**A**^	323.9*	11.2	322.2	301–347	295–305 mmol/kg
**ST** ^**B**^	316.8†	9.6	313.8	296–333	290–300 mmol/kg
**Osmolal Gap**^**C**^	6.71	7.3	4.53	0.14–39.49	≤10 mmol/kg
**Tonicity Gap**^**D**^	8.10	9.07	4.90	0.34–47.34	≤10 mmol/kg
**dMCV**	2.74	1.50	2.97	-0.69–5.75	

^A^ OsM_C_ = 2(Na + K) + Glucose + SUN

^B^ ST = 2(Na + K) + Glucose

^C^ Osmolal gap = OsM_T_−OsM_C_

^D^ Tonicity gap = OsM_T_−ST

* No significant difference; p = 0.93.

^†^Significant difference; p = 0.0004.

Summary statistics for serum concentrations of major osmoles (Na, K, glucose, SUN), osmolality assessments (OsM_T_, OsM_C_, and ST) and dMCV from dogs stratified by serum glucose concentration are shown in Table 2. Nearly all dogs had hyperglycemia (87.5%) but euglycemia was present in 3 dogs. A single dog had hypoglycemia (3.2 mmol/L), which was subclinical. Among those with dysglycemia, serum glucose was ≥ 13.9 mmol/L in approximately 60% of dogs. Fifteen dogs had serum glucose concentrations < 13.9 mmol/L and 17 dogs had clinically relevant hyperglycemia with serum glucose concentrations ≥ 13.9 mmol/L. Glucose concentrations in the latter group ranged from 14–29.1 mmol/L. Serum Na and SUN did not differ between the groups but there was a small but statistically significant difference in serum K between the groups (p = 0.0078). Dogs with serum glucose ≥ 13.9 mmol/L had significantly higher OsM_T_ (p = 0.0004) and increased dMCV (p = 0.0047) compared with dogs with serum glucose <13.9 mmol/L.

**Table 2:** Summary statistics for dogs stratified by serum glucose concentration.

	Serum glucose < 13.9 mmol/L (n = 15)		Serum glucose ≥ 13.9 mmol/L (n = 17)		
	Mean (+/- SD)	Median(Range)	Mean (+/- SD)	Median(Range)	Reference Interval
Sodium (Na) (mmol/L)	146.5 (3.5)	146 (141–154)	145.0 (3.43)	145 (139–152)	144–151
Potassium (K) (mmol/L)	4.6 (0.38)*	4.5 (3.9–5.3)	5.04 (0.47)*	5.1 (3.9–5.8)	3.7–5.0
Serum Urea Nitrogen (SUN) (mmol/L)	6.1 (3)	5 (2.5–11.8)	7.8 (2.8)	7.9 (4.3–15.7)	2.86–10.34
Glucose (mmol/L)	8.3 (3.0)	8.6 (3.2–12.67)	22.4 (5.0)	24.3 (14–29.1)	3.89–6.67
OsM_T_ (mmol/kg)	315.2 (7.4) ^†^	317 (302–325)	331.5 (14.8) ^†^	330 (315–376)	
OsM_C_^A^ (mmol/kg)	310 (7.5)	312 (296–322)	322.5 (7.6)	326 (310–333)	
ST ^B^ (mmol/kg)	317 (9.0)	315 (301–333)	330.3 (8.8)	331 (348–315)	
Osmolal Gap^C^ (mmol/kg)	6.0 (4.25)	5.58 (0.45–12.6)	7.3 (9.3)	4.3 (0.14–39.5)	<10
Tonicity Gap^D^ (mmol/kg)	6.6 (5.6)	4.6 (0.34–17)	9.4 (11.3)	5.4 (1.1–47.3)	<10
dMCV (μm^3^)	1.99 (1.24) ^+^	1.87 (-0.7–3.8)	3.41 (1.4) ^+^	3.77 (0.38–5.75)	<3

^A^ OsM_C_ = 2(Na + K) + Glucose + SUN

^B^ ST = 2(Na + K) + Glucose

^C^ Osmolal gap = OsM_T_−OsM_C_

^D^ Tonicity gap = OsM_T_−ST

* Significant difference; p = 0.0078.

^†^Significant difference; p = 0.0004.

+Significant difference; p = 0.0047

**Fig 1:**
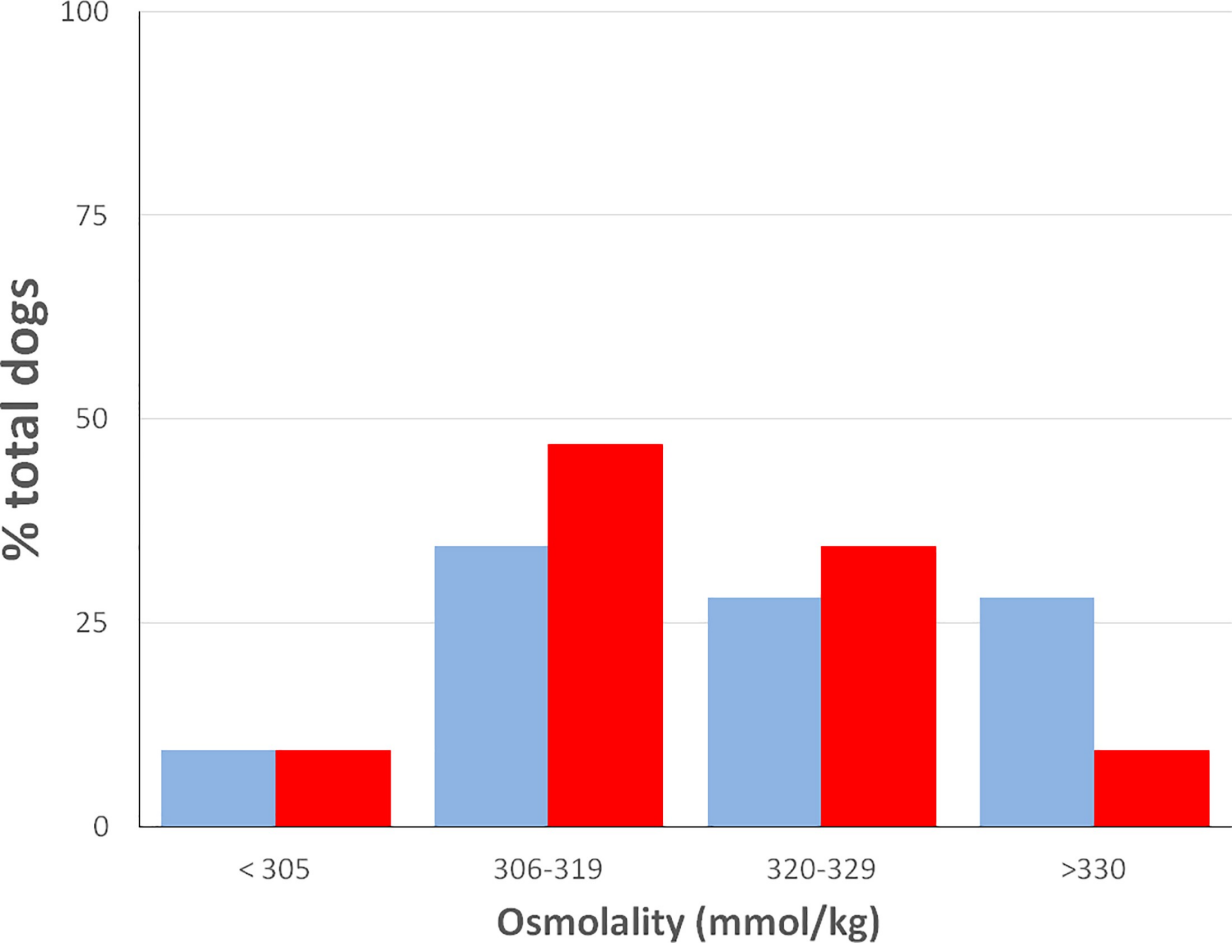
Classification of dogs by serum osmolarity or serum tonicity. Classification of diabetic dogs as having normal tonicity (< 305 mmol/kg), mild hypertonicity (305–319 mmol/kg), moderate hypertonicity (320–329 mmol/kg), and severe hypertonicity (≥ 330 mmol/kg) using serum total measured osmolality (blue bars) or calculated tonicity (red bars). The effect of urea and other ineffective osmoles on total osmolality results in overestimation of the prevalence of severe hypertonicity in diabetic dogs.

The mean serum OsM_T_ (323 mmol/kg) for all dogs was approximately 8% increased over the ‘normal’ osmolar set point of dogs, which is generally accepted to be 300 +/- 5 mmol/kg [21]. The majority of dogs (91%) had hyperosmolality (Fig 1). Only three dogs (9%) had normal osmolality (OsM_T_ ≤ 305 mmol/kg). Of the remainder, 34% of dogs had an OsM_T_ between 306–319 mmol/kg (mild hyperosmolality), 28% of dogs had an OsM_T_ between 320–329 mmol/kg (moderate hyperosmolality), and 28% of dogs had an OsM_T_ ≥ 330 mmol/kg (severe hyperosmolality).

**Fig 2:**
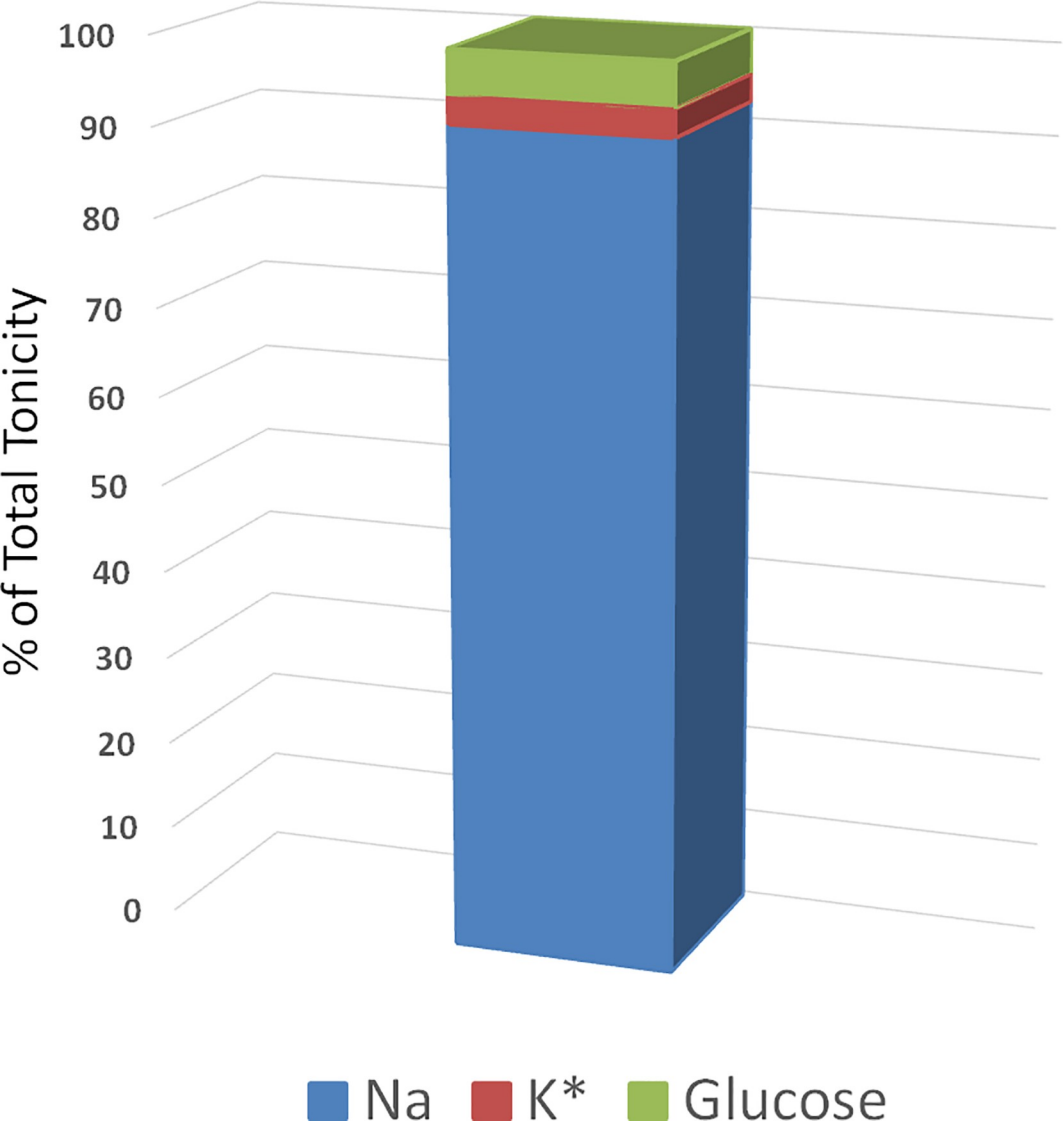
Contribution of measured effective osmoles to serum tonicity in insulin-treated diabetic dogs. Glucose (green bar) contributed 5% of effective osmoles to serum tonicity in insulin-treated diabetic dogs. Sodium (blue bar) provided the majority of effective osmoles and with potassium (red bar) contributed a combined 95% of effective osmoles to serum tonicity. For simplicity, osmoles contributed by sodium and its corresponding anions and by potassium and its corresponding anions are represented, respectively, as ‘sodium’ and ‘potassium’ in the figure.

Mean ST for all dogs was 316 mmol/kg (normal tonicity = 295 +/- 5 mmol/kg [15, 21]) and hypertonicity was present in 94% of dogs (Fig 1). Only 2 dogs had serum tonicity in the normal range. Mild hypertonicity (306–319 mmol/kg) was present in 47% of diabetic dogs. Moderate hypertonicity (320–329 mmol/kg) was present in 34% of dogs and severe hypertonicity (≥ 330 mmol/kg) in 9.4% of dogs. Overall, fourteen dogs (44%) had a ST ≥ 320 mmol/kg, indicating that clinically relevant hypertonicity was prevalent in the study dogs. Classification by ST and OsM_T_ was also carried out after dogs were first stratified by whether or not clinically relevant hyperglycemia (serum glucose ≥ 13.9 mmol/L) was present (Table 3). Clear differences were observed between the groups. None of the dogs with serum glucose ≥ 13.9 mmol/L (n = 17) had ST within the reference range (< 305 mmol/kg) compared with 20% of dogs with serum glucose < 13.9 mmol/L. Conversely, 70.5% of dogs with serum glucose ≥ 13.9 mmol/L had a ST > 320 mmol/kg compared with just 13.3% of dogs with serum glucose <13.9 mmol/L, indicating that the largest proportion of study dogs with clinically relevant hypertonicity also had clinically relevant hyperglycemia. Analysis of the proportional contribution of major effective osmoles to serum tonicity in all dogs showed that sodium contributed 92%, potassium 3%, and glucose 5% of serum effective osmoles (Fig 2).

**Table 3:** Serum tonicity and serum osmolarity in dogs stratified by serum glucose concentration ≥ 13.9 mmol/L (n = 17) or serum glucose concentration < 13.9 mmol/L (n = 15).

	≤305 mmol/kg		306–319 mmol/kg		320–329 mmol/kg		≥330 mmol/kg	
	ST	OsM_M_	ST	OsM_M_	ST	OsM_M_	ST	OsM_M_
Glucose ≥13.9 mmol/L	0%	0%	29.4%	23.5%	52.9%	23.5%	17.6%	52.9%
Glucose <13.9 mmol/L	20%	20%	66.7%	46.7%	13.3%	33.3%	0%	0%

Data shown represents % of cases in each stratified group that are included under each classification.

**Fig 3:**
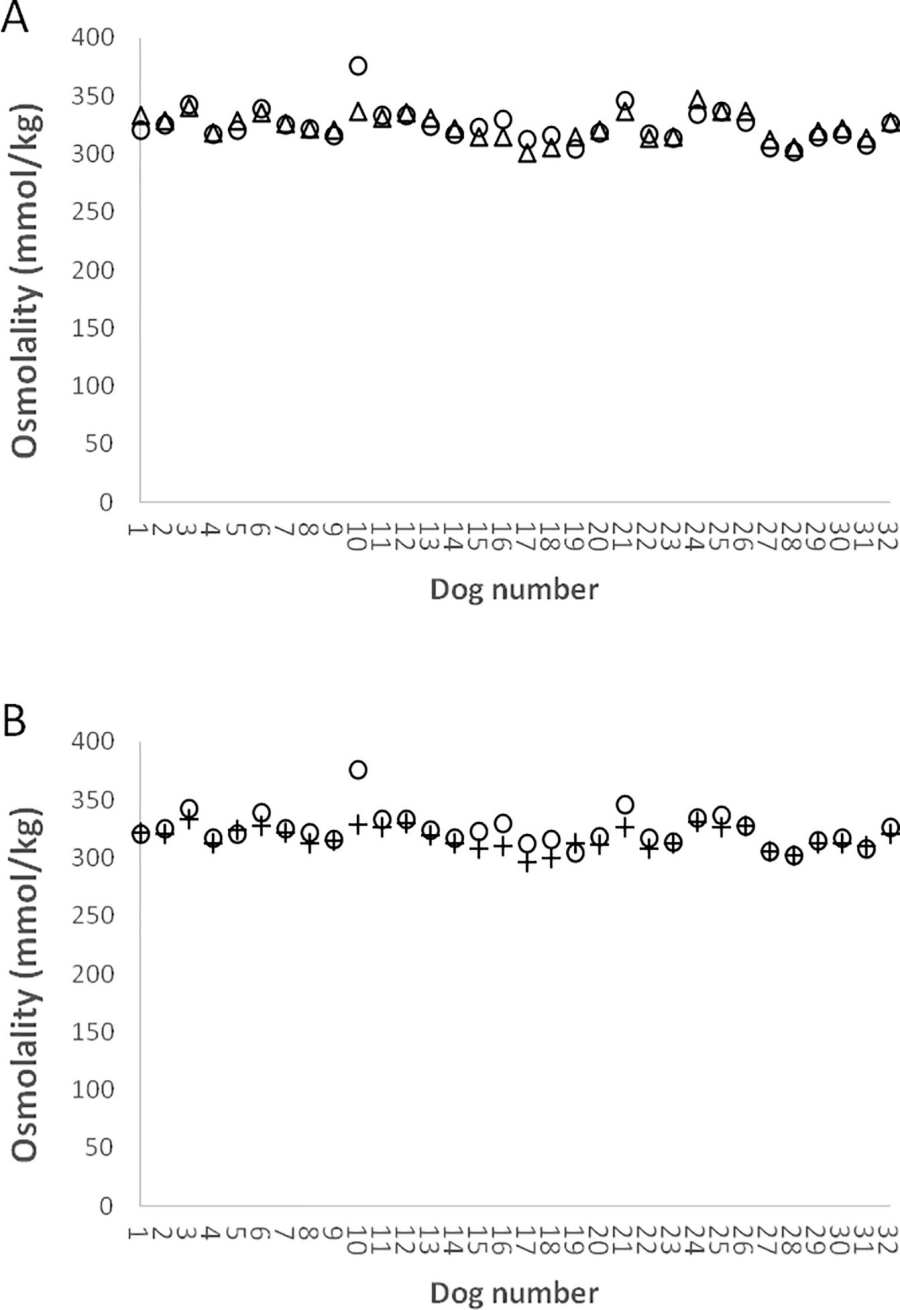
Comparisons between OsM_T_ and OsM_C_ and OsM_T_ and ST. Panel A–Serum OsM_T_ (open circles) and serum OsM_C_ (open triangles) of individual dogs (n = 32) are shown. The mean osmolal gap (difference between measured and calculated total osmolality) was normal (< 10 mmol/kg). Mean OsM_T_ and mean OsM_C_ were not significantly different (p = 0.93). Panel B—OsM_T_ (open circles) and calculated ST (crosses) of individual dogs (n = 32) are shown. The mean tonicity gap (difference between OsM_T_ and calculated ST) was <10 mmol/kg. OsM_T_ and calculated ST were significantly different (p = 0.0004).

Serum OsM_T_ compared favorably to the OsM_C_ and ST (Fig 3). Serum OsM_T_ did not differ from the serum OsM_C_ (p = 0.93) but differed significantly from ST (p = 0.0004). Over 50% of dogs with serum glucose ≥ 13.9 mmol/L had a serum OsM_T_ > 330 mmol/kg, yet less than 20% had ST >330 mmol/kg (Table 2). The presence of ineffective osmoles not considered in the determination of ST, such as SUN, likely contributed to the OsM_T_ in this group. The mean osmolal gap (6.7 mmol/kg) was within the expected range (≤ 10 mmol/kg) for dogs [21]. The mean tonicity gap between the OsM_T_ and ST (8.1 mmol/kg) was slightly larger but also within the expected range.

**Fig 4:**
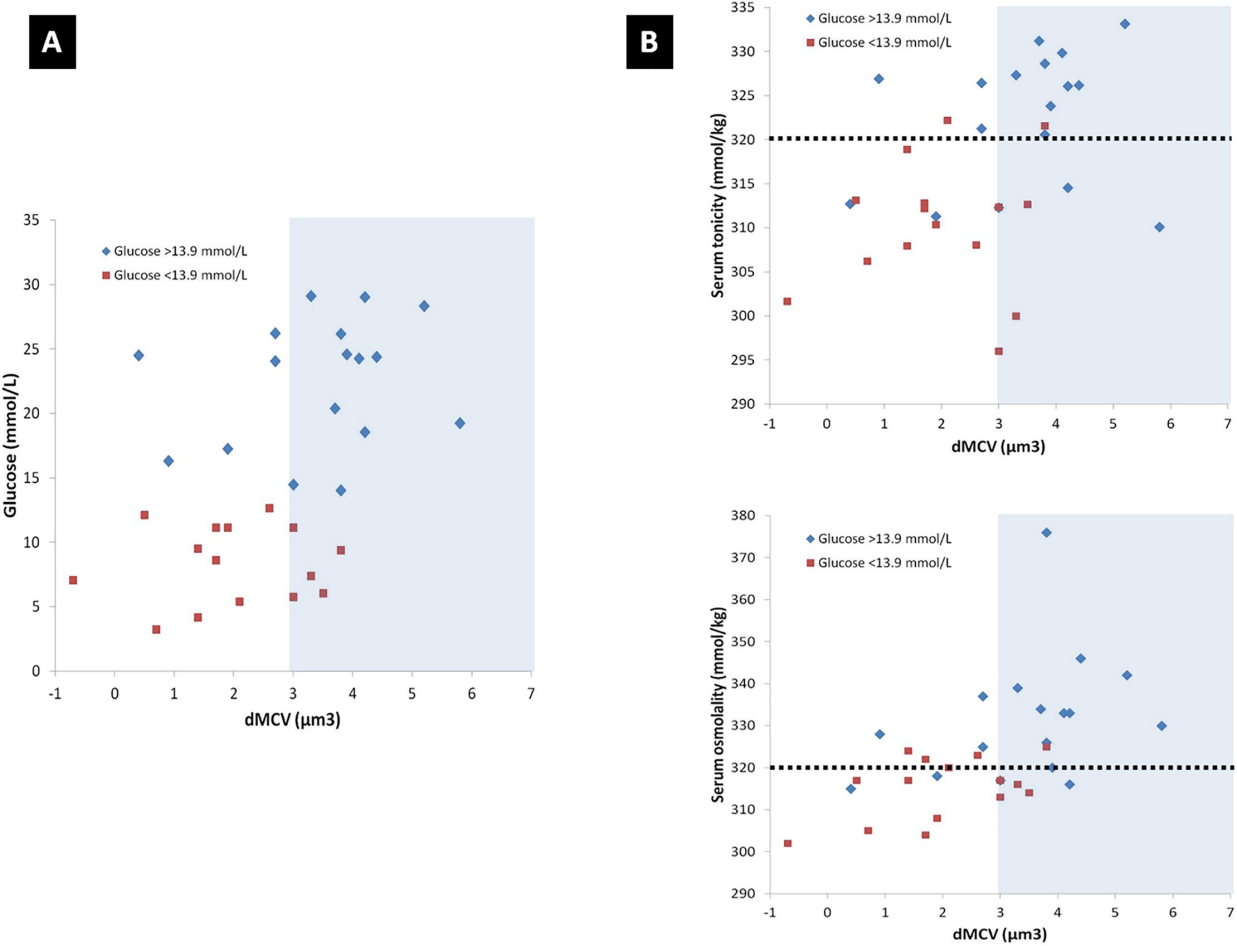
Relationships between dMCV and glucose, ST, and OsM_T_. Panel A–The relationship between the dMCV and serum glucose in dogs with serum glucose concentration ≥ 13.9 mmol/L (blue diamonds) or < 13.9 mmol/L (red squares) is shown. The blue shaded area indicates dMCV values above the 3 μm^3^ cut-off used to classify dogs with serum hypertonicity. Panel B–Relationships between dMCV and ST (top) and OsM_T_ (bottom) in dogs classified as described in Panel A. The blue shaded area indicates dMCV values above the 3 μm^3^ cut-off used to classify dogs with serum hypertonicity. The black dotted line indicates the cut-off (320 mmol/kg) that represents clinically relevant elevations in ST and OsM_T_.

The mean dMCV was 2.74 μm^3^. When dogs were stratified according to whether or not clinically relevant hyperglycemia was present, the dMCV in dogs with high glucose (3.41 μm^3^) was significantly larger than in the group with lower glucose (1.99 μm^3^) (Table 3). Fifty-three percent of dogs were classified as having serum hypertonicity using a previously determined dMCV cut-off value of ≥ 3 μm^3^ [6]. The dMCV was significantly correlated to the serum glucose, ST, and OsM_T_ (Fig 4). Correlations between dMCV and glucose (R = 0.49; p = 0.0042) and dMCV and OsMT (R = 0.55; p = 0.0111) were moderate, while dMCV and ST showed a slightly weaker correlation (R = 0.43; p = 0.0133). Among dogs with dMCV ≥ 3 μm^3^, 70% had a serum glucose ≥ 13.9 mmol/L. In contrast, just 33% of dogs with serum glucose < 13.9 mmol/L had a dMCV ≥ 3 μm^3^. Similarly, 86% of dogs with ST ≥ 320 mmol/kg and 72% of dogs with OsMT ≥ 320 mmol/kg also had serum glucose ≥ 13.9 mmol/L. Using the 3 μm^3^ cut-off, dMCV had 79% sensitivity and 61% specificity for detecting clinically relevant serum hypertonicity (ST ≥ 320 mmol/kg). Diabetic dogs with dMCV ≥ 3 μm^3^ had increased risk for having serum hypertonicity (ST ≥ 320 mmol/kg; RR 2.2; p = 0.0158) and for hyperglycemia (GLU ≥ 13.9 mmol/L; RR 2.3; p = 0.0329). In contrast, a dMCV ≥ 3 μm^3^ did not convey risk for hyperosmolality (OsM_T_ ≥ 320 mmol/kg; RR 1.56; p = 0.2078).

## Discussion

Previous investigations that examined dMCV as a hypertonicity marker in dogs were carried out using normal dogs or dogs with non-diabetic illness in which hypertonicity was primarily due to sodium disturbances [18, 19]. The previous studies did not specifically examine the usefulness of dMCV for predicting serum hypertonicity when glucose is the primary metabolic disturbance (i.e. hyperglycemia). In the current study, we now show that the dMCV is increased in diabetic dogs with hypertonicity caused by hyperglycemia. Furthermore, the findings show that diabetic dogs with an increased dMCV are more likely to have clinically relevant increases in glucose and serum tonicity. The results affirm the usefulness of dMCV as a clinical marker for hypertonicity in dogs with diabetes mellitus when the predominant increase in tonicity is due to hyperglycemia.

Erythrocyte changes, including an increase in the mean cell volume (MCV), are known to occur in diabetic dogs and may reflect effects of the disease on various aspects of erythrocyte metabolism [22]. The dMCV, the focus of the current study, represents the difference in MCV that occurs when erythrocytes from a hypertonic patient undergo an osmotic change after exposure to the isotonic buffer (isotonic to normal plasma) used by automated hematology analyzers. In healthy adult dogs undergoing 24-hour water deprivation, increased dMCV was noted along with increases in serum sodium, serum total osmolality, and urine osmolality [19]. The influence of specific patient factors, such as gender, age, or reproductive status, on erythrocyte responses and the dMCV has not been examined. However, the dMCV appears to be useful when applied to unselected patient populations. In a group of hospitalized dogs with a variety of illnesses, a dMCV value ≥ 3 μm^3^ had sensitivities that ranged from 67 to 76% and specificities that ranged from 68 to 71% depending on whether OsM_T_, OsM_C_, or ST was used to classify the dog as hypertonic [18]. In the current study, the same cut-off value for dMCV had similar sensitivity and specificity when dogs were classified using ST, suggesting that dMCV is a useful marker for serum hypertonicity in diabetic dogs.

Disturbances of serum osmolality are frequently encountered in complicated diabetes, such as diabetic ketoacidosis and hyperosmolar hyperglycemic syndrome. Hyperosmolarity was common among untreated dogs with non-ketonuric or ketonuric diabetes or the hyperosmolar hyperglycemic syndrome in several studies [6, 15]. Hyperosmolality is important to understand because it is associated with risk for osmotic complications in diabetic patients, including severe neurologic dysfunction, which may be fatal [23, 24, 25]. The frequency and range of osmotic complications due to hyperosmolality and, more specifically, hypertonicity in dogs are currently unknown. Unfortunately, veterinary studies often fail to highlight the distinction between total hyperosmolality, either measured or calculated, and tonicity. As a result, tonicity is frequently overestimated when its value is inferred from total osmolality (calculated or measured), especially when azotemia or acidosis are present [6,15]. Thus, a specific pathophysiologic role for tonicity remains difficult to define in diabetic dogs. In the current study, more than half of dogs had serum OsM_T_ ≥ 320 mmol/kg, which indicates moderate to severe osmolar disturbances were common in this population. One dog, which had been assessed as neurologically normal, had an OsM_T_ = 376 mmol/kg, but the OsM_C_ and ST, respectively, were 337 and 329 mmol/kg. Although it is likely that the measured osmolality for this dog represented a laboratory error, the osmometry result met the criteria for acceptance and was included in all analyses. Since azotemia and hypernatremia were not prevalent in the study group, the observed elevations in total osmolality are due to hyperglycemia. This conclusion is supported by the finding that the osmolal gap, which is increased when unmeasured osmoles are present in serum, was normal in the study dogs. Nevertheless, when dogs were categorized using ST (Fig 1), fewer dogs were classified as having severe hyperosmolality than when categorized based on OsM_T_. This observation, along with the finding of a significantly larger osmolal gap between OsM_T_ and ST, an expected finding because SUN is not included when estimating serum tonicity, suggests that reliance on measured total osmolality may overestimate tonicity in insulin-treated, clinically stable diabetic dogs as it does in dogs with complicated diabetes [6,15]. Thus, in the stable diabetic dog, evaluation of the dMCV in combination with with the calculated ST may be more useful that OsM_T_ to establish whether a patient has serum hypertonicity.

Nearly all of the diabetic dogs in this outpatient population had some degree of serum hypertonicity. Interestingly, the prevalence of clinically relevant hypertonicity in the insulin-treated dogs reported here is similar to the prevalence observed in untreated dogs with diabetic ketosis [6, 26], suggesting that chronic hypertonicity may persist despite insulin therapy and constitute a concern in long-term management of diabetic dogs.

Hypertonicity in insulin-treated diabetic dogs in this study developed secondary to hyperglycemia and as such likely reflects suboptimal glycemic control. Additional studies that provide a quantitative index of overall glucose control are needed to provide greater insight into the cause and consequences of serum hypertonicity in treated diabetic dogs. Based on our findings that over 50% of dogs had serum glucose greater than 13.9 mmol/L and of that group, 60% had severe hyperglycemia (> 22.2 mmol/L), it is likely that suboptimal glycemic control was common among the study group.

The increase in serum tonicity in the diabetic dogs was due to hyperglycemia, which persisted despite insulin therapy. Tonicity is dependent on the number rather than the chemical identity of the effective osmoles that are in solution. In dogs with euglycemia, glucose contributes relatively few osmoles to serum tonicity and sodium is the major effective osmole. However, the role of glucose is substantially greater in diabetes. In the current study, glucose contributed an average of 5% of serum effective osmoles. A similar increase in the relative contribution of glucose to serum tonicity has been reported in untreated cats with diabetic ketoacidosis [7] and in another group of diabetic dogs [26]. The importance of modest hyperglycemia for producing a hypertonic state in the treated diabetic dog can be understood in the context of current diabetes management practices. A mean 24-hour glucose concentration that is less than the renal threshold for glucosuria is frequently cited as a desired target for effective insulin treatment in diabetic dogs [27]. Dogs with an average daily glucose concentration < 10 mmol/L display minimal clinical signs and are usually considered to have adequate glycemic control [28]. However, since glucose maintained at the therapeutic target contributes the equivalent of 10 mmol/kg to serum osmolality, even some “well controlled” diabetic dogs may experience chronic hypertonicity and the effect is expected to be more pronounced in dogs with unacceptable glycemic variability due to glucose fluctuations during the day or in dogs with suboptimal overall diabetic control.

Currently, the importance of serum hypertonicity among clinically stable, diabetic dogs is not understood. Certain hyperglycemic signs, such as marked thirst, are mediated in part via mechanisms related to hypertonicity. Hypertonicity also influences *in vivo* insulin release in healthy men given a saline challenge [29] and *in vitro* insulin secretion in beta cell or islet preparations [30]. Along with these well-established actions on insulin release, it is possible that hypertonicity exerts other, more subtle, effects on glucose metabolism. For example, among humans at risk for the development of diabetes, hypertonicity conveys risks for complications that are independent of hyperglycemia [11, 31].

Limititations of the design and study population should be considered in interpreting the study results. In the current study, the dMCV was about 80% sensitive and 60% specific for detecting hypertonicity, which suggests it is less useful than the ST calculated using the concentrations of measured effective serum osmoles, which served as the gold standard for the current study. In comparing these two methods, it should be recognized that both have limitations. Although the equation used to obtain ST lends the appearance of an accurate and precise assessment, in fact it simply yields an estimate of tonicity. Calculated ST can also be affected by fluctuations in the serum glucose, which occur over the course of the day in diabetic dogs, especially those with less than optimal glycemic control. Thus, ST determined using a spot glucose determination could provide inaccurate information about magnitude and duration of hypertonicity. The calculated ST is an imperfect gold standard since it does not consider possible contributions from unmeasured effective osmoles nor does it reflect the influences of physiological mechanisms that may become relevant when multiple disturbances in effective osmole concentrations are present (e.g. the diabetic dog with changes in glucose, sodium, and potassium). In contrast, the dMCV, a dynamic measure of a cellular response, may better reflect the physiologic processes involved in the chronic adaptation to serum hypertonicity. Other advantages of the dMCV over the calculated ST may lie in its potential for use in screening studies where a convenient sample is preferred and its sensitivity for detecting very slight changes in serum tonicity, even when measured osmoles (sodium, glucose, and others) remain in the reference range [19]. The effects of acute changes in glucose (or other osmoles) on the dMCV have not been studied and the relationship of the dMCV response to the duration of hypertonicity is unknown. Characteristics of the study population should be considered when interpreting the results. The study enrolled a non-selected patient population and the sample size was small. Insulin-treated diabetic dogs that were considered as clinically stable and maintained as outpatients were enrolled but participation was not based on any objective criteria that might be associated with overall glucose control, such as mean daily glucose concentration, serum fructosamine, blood hemoglobin A1c, or clinical signs and no attempt was made to objectively classify glucose control prior to enrollment. In this study, a non-selected enrollment was used to ensure a clinically-relevant cross section of diabetic dogs were represented in the study group. This design has additional advantages in that study enrollment goals are met sooner and a higher proportion of diabetic dogs are eligible for participation. However, it is possible that the dMCV might perform differently in a patient population that was more stringently selected (based on glycemic control, for example) or in a study with a larger enrollment.

In conclusion, the usefulness of the dMCV for detecting hypertonicity in diabetic dogs was demonstrated using an outpatient population. In insulin-treated, clinically stable diabetic dogs, an elevated dMCV was associated with significant risk for moderate to severe hypertonicity (ST ≥ 320 mmol/kg) and moderate to severe hyperglycemia (glucose ≥ 13.9 mmol/kg) but was not associated with risk for moderate to severe increases in measured serum total osmolality (OsM_T_ ≥ 320 mmol/kg). The finding is consistent with expectations that changes in the dMCV reflect erythrocyte adaptations to elevated serum concentrations of effective osmoles in diabetes. Taken together, our findings show that the dMCV is a useful surrogate marker for detecting clinically relevant serum hypertonicity in diabetic dogs.

## Supporting information

S1 File Norris data archive. Spreadsheet containing clinical data for study dogs.
(XLSX)
