# Correction: The mean cell volume difference (dMCV) reflects serum hypertonicity in diabetic dogs

**DOI:** 10.1371/journal.pone.0222820

**Published:** 2019-09-16

**Authors:** Olga C. Norris, Thomas Schermerhorn

The following information is missing from the Funding statement: Publication of this article was funded in part by the Kansas State University Open Access Publishing Fund.

In the Methods section, there is a typographical error in equation 3. Please view the complete, correct equation here:

$$dMCV={MCV}_{M}-(hct\times 10/RBC)$$
